# Supplementary material for: Adaptation of White Adipocytes to Cooler Temperatures: Impacts on Energy Metabolism and Protein Acetylation
Source: FASEB J. 2026 Aug 2;40(15):e72164. doi: 10.1096/fj.202601941R (PMC13430111; doi:10.1096/fj.202601941R)
Supplement: Supplementary file 1 — Figure S1: The breast cancer cell line MDA‐MB‐231 was incubated at 31°C for the indicated durations. Whole‐cell lysates were analyzed by immunoblotting for acetylated lysine, with laminin, HSP70, and HSP90. Figure S2: Differentiated adipocytes were cultured at either 37°C or 31°C for 10 days, followed by treatment with 20–100 μM nicotinic acid for 2 days. Figure S3: Representative MS/MS spectrum of the acetylated PCCA peptide AK[Acetyl (K)]VNTIPGFDGVVK, TFDK[Acetyl (K)]ILIANR. Blue and red peaks indicate y‐ and b‐ions, respectively, and the fragment pattern supports localization of acetylation to the indicated lysine residue. Figure S4: Representative MS/MS spectrum of the acetylated SHMT2 peptide LQDFK[Acetyl (K)]SFLLK, TAK[Acetyl (K)]LQDFK, TGK[Acetyl (K)]EIPYTFEDR, and YSEGYPGK[Acetyl (K)]R. Blue and red peaks indicate y‐ and b‐ions, respectively, and the fragment pattern supports localization of acetylation to the indicated lysine residue. [file FSB2-40-e72164-s001.docx]

**Supplementary figures**

**Adaptation of white adipocytes to cooler temperatures: impacts on energy metabolism and protein acetylation**

Hiroyuki Mori^1, #^, Hadla Hariri^1, #^, William Moe^1^, Sophia Durham^1^, Yuridia Guzman^1^, Emma Paulsson^1^, Rachel C. Simmermon^1^, Parth B. Bhanderi^1^, Sydney K. Peterson^1^, Mia J Dickson, Charles R. Evans^2^, Ormond A. MacDougald^1,2^

**Supplemental Figure 1.** The breast cancer cell line MDA-MB-231 was incubated at 31°C for the indicated durations. Whole-cell lysates were analyzed by immunoblotting for acetylated lysine, with laminin, HSP70, and HSP90.

**Supplemental Figure 2.** Differentiated adipocytes were cultured at either 37°C or 31°C for 10 days, followed by treatment with 20–100 μM nicotinic acid for 2 days.


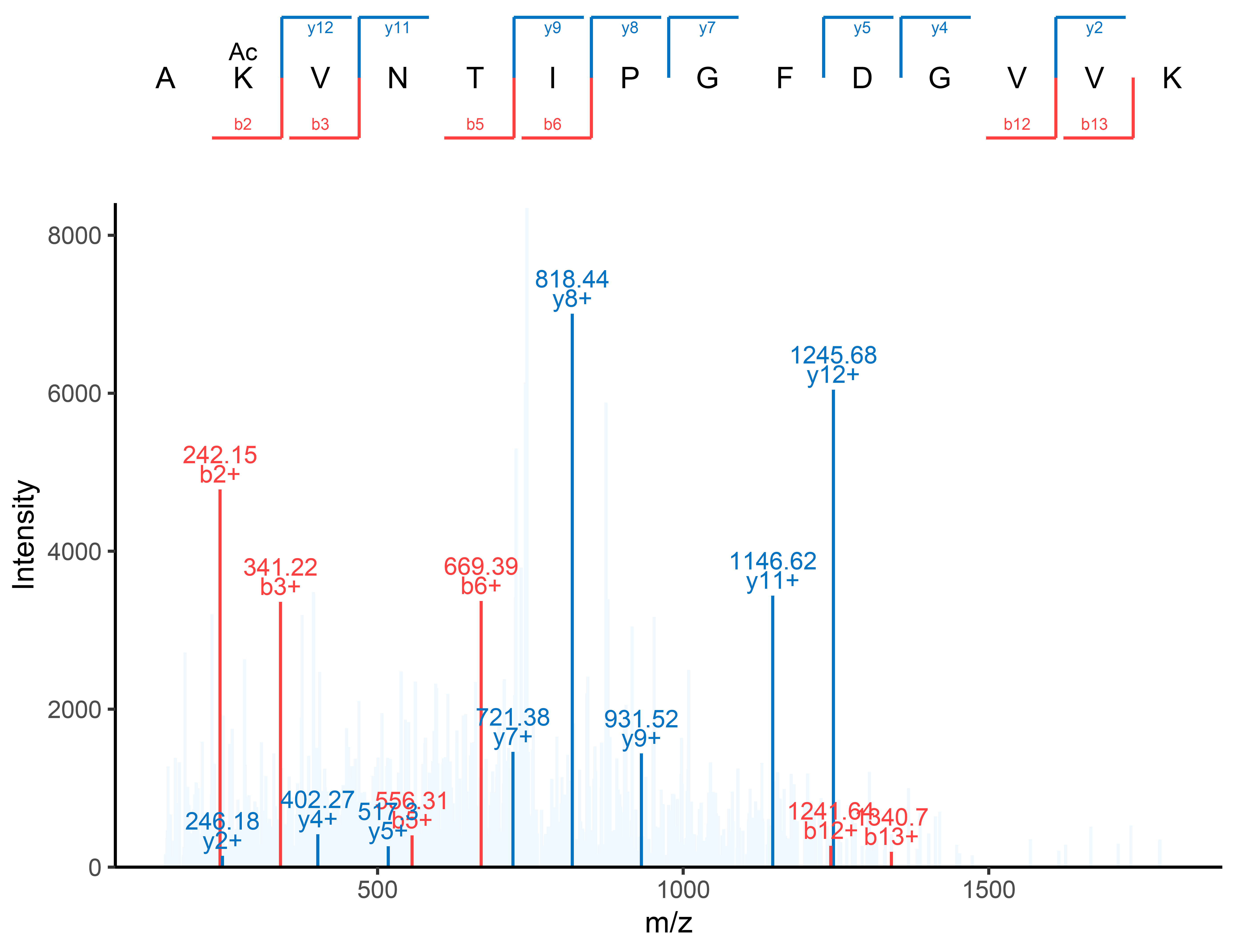

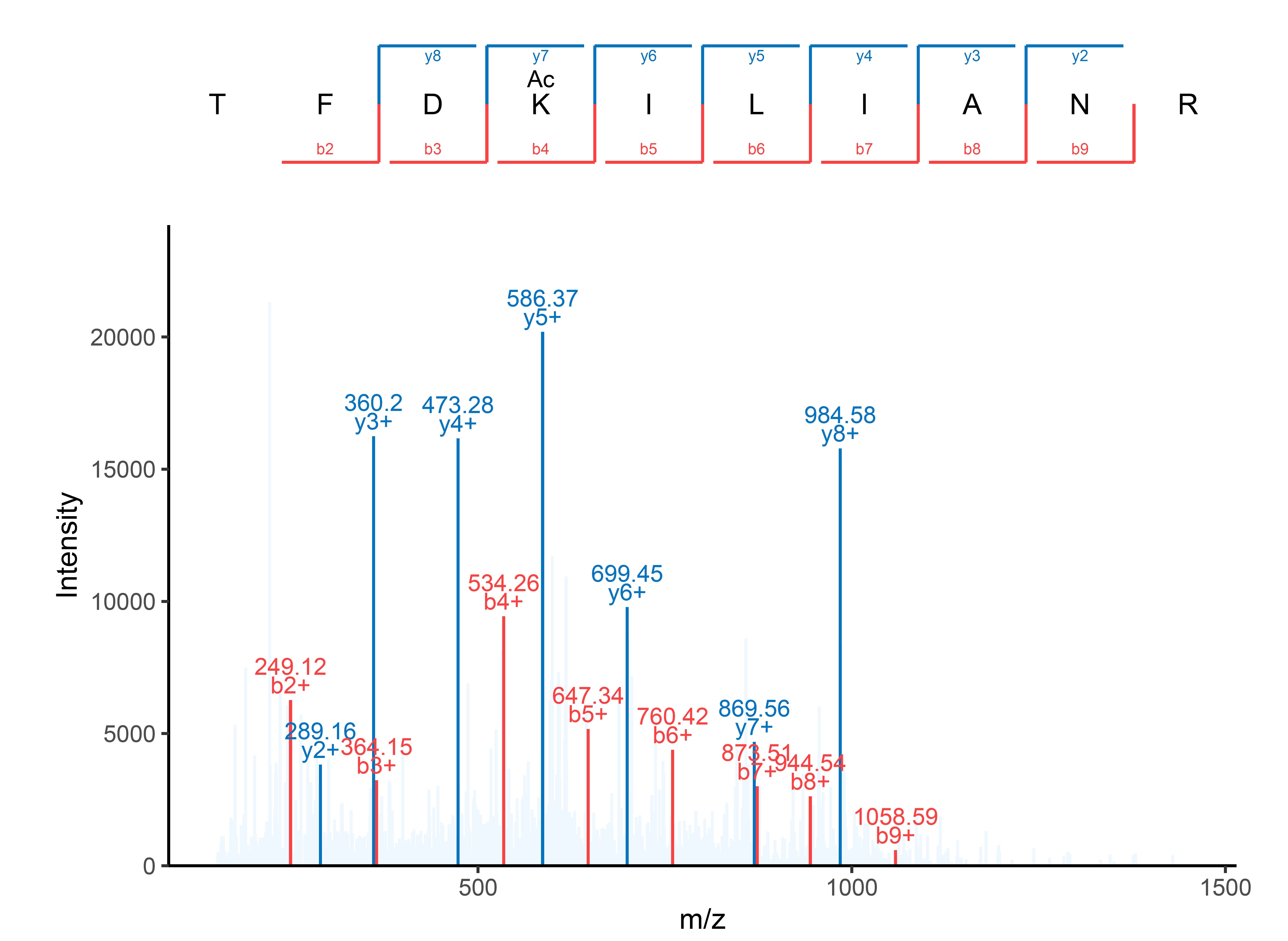


**Supplemental Figure 3.**

Representative MS/MS spectrum of the acetylated PCCA peptide AK[Acetyl (K)]VNTIPGFDGVVK, TFDK[Acetyl (K)]ILIANR. Blue and red peaks indicate y- and b-ions, respectively, and the fragment pattern supports localization of acetylation to the indicated lysine residue.


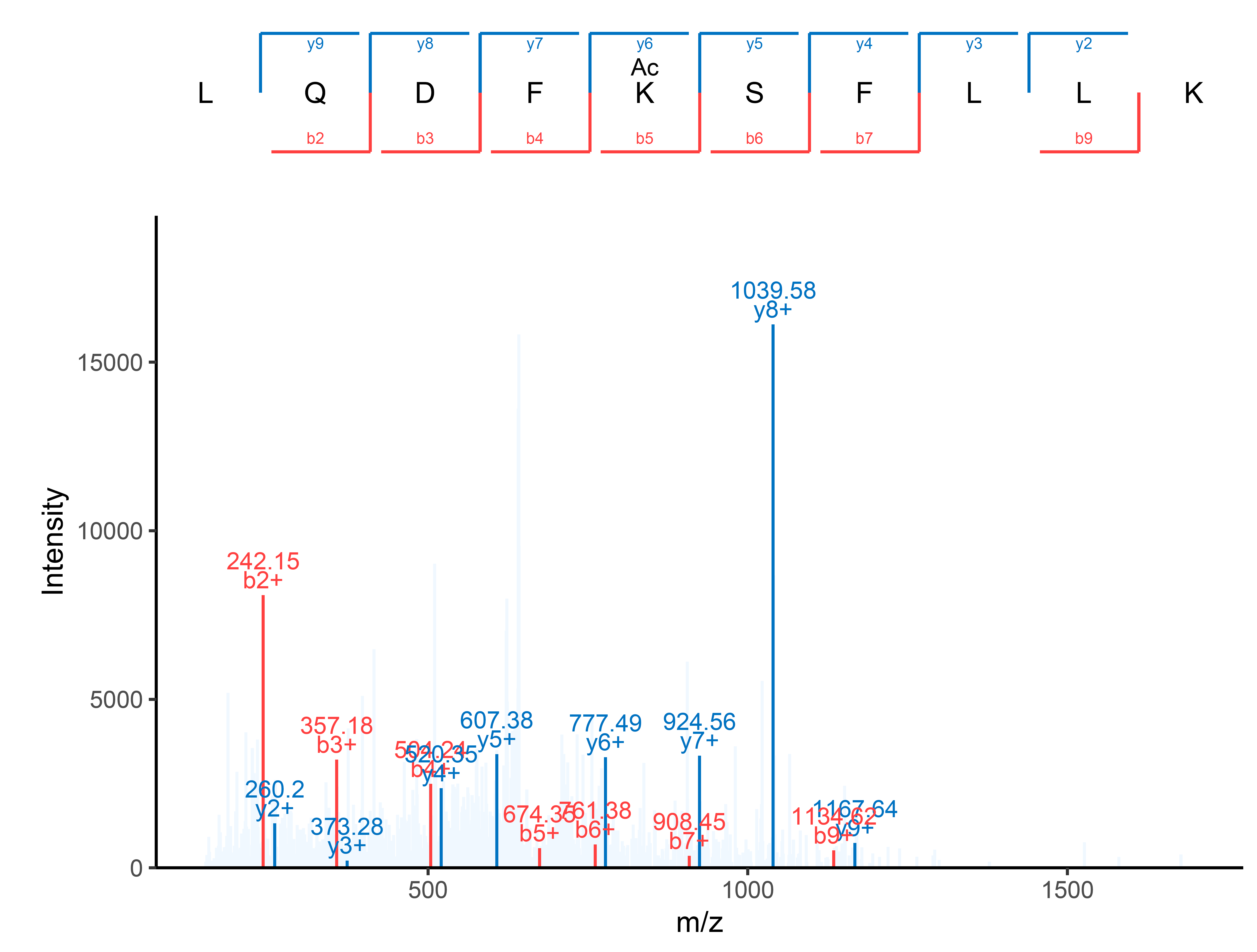

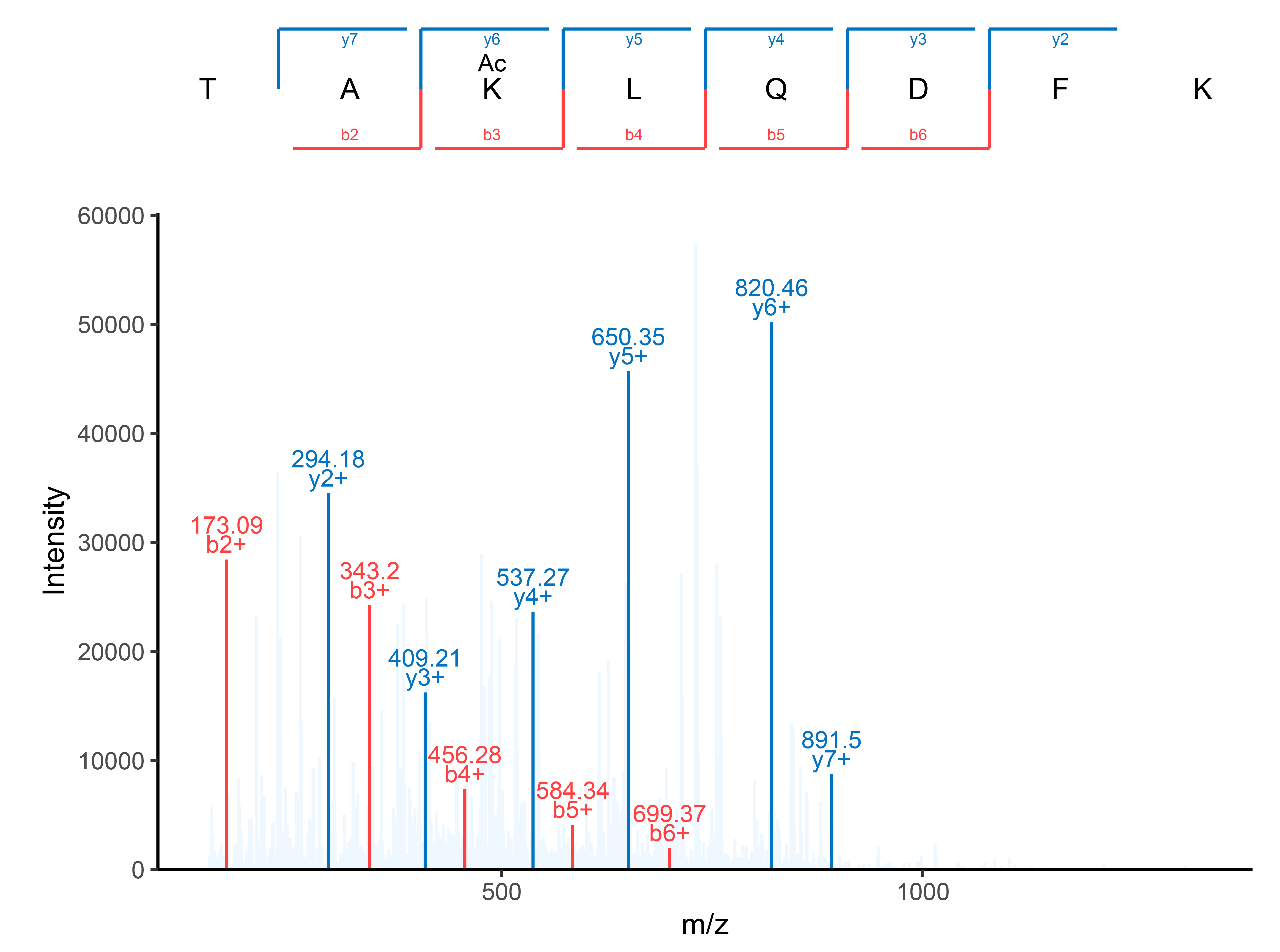


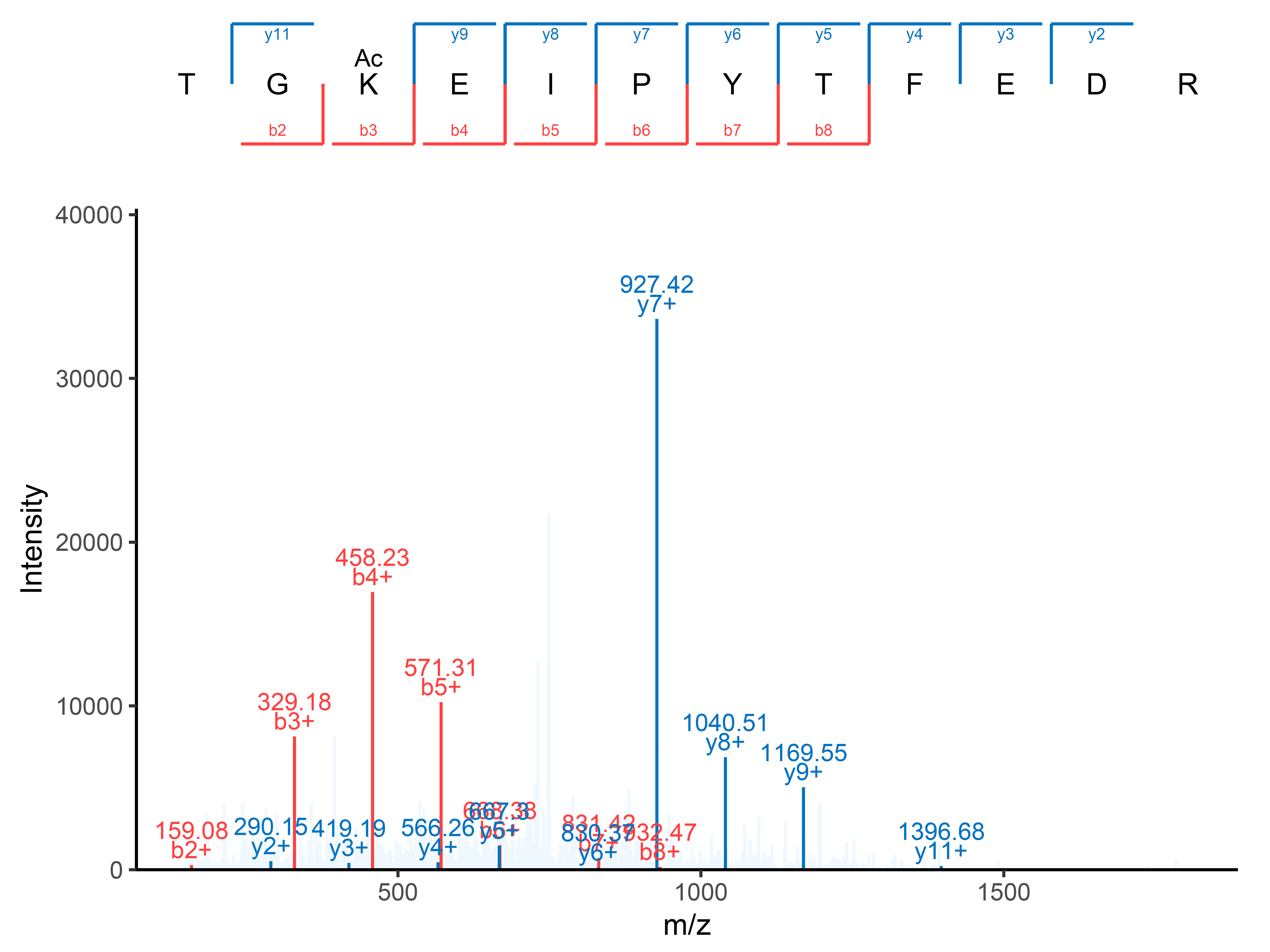

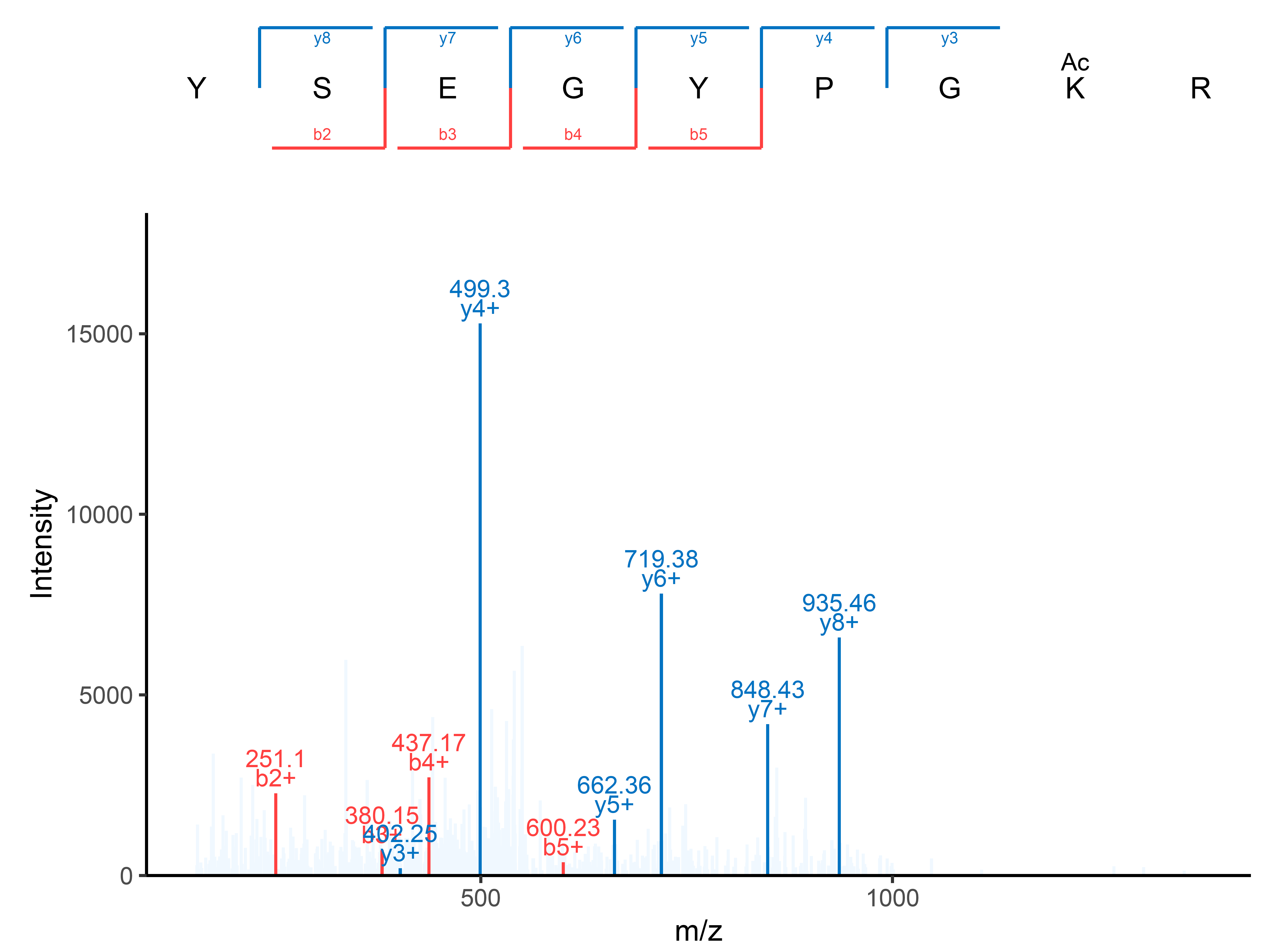


**Supplemental Figure 4.**

Representative MS/MS spectrum of the acetylated SHMT2 peptide LQDFK[Acetyl (K)]SFLLK, TAK[Acetyl (K)]LQDFK, TGK[Acetyl (K)]EIPYTFEDR, and YSEGYPGK[Acetyl (K)]R. Blue and red peaks indicate y- and b-ions, respectively, and the fragment pattern supports localization of acetylation to the indicated lysine residue.
